# Molecular Investigation of *Anaplasma* spp. and Genotype Profile of *A. ovis* in Sheep from Different Farms in Türkiye

**DOI:** 10.1007/s11686-025-01021-2

**Published:** 2025-04-10

**Authors:** Özge Dülek, Çağrı Kandemir, Ecem Su Koçkaya, Ecem Sürgeç, Mervenur Güvendi, Muhammet Karakavuk, Aysu Değirmenci Döşkaya, Özlem Günay-Esiyok, Turgay Taşkın, Mert Döşkaya, Cemal Ün, Adnan Yüksel Gürüz, Ahmet Efe Köseoğlu, Sedef Erkunt Alak, Erkan Pehlivan, H. Deniz Şireli, Serdar Koçak, Ömer Faruk Yılmaz, Hüseyin Can

**Affiliations:** 1 Department of Biology Molecular Biology Section, Faculty of Science, Ege University, İzmir, Türkiye; 2 Department of Animal Science, Faculty of Agriculture, Ege University, İzmir, Türkiye; 3 Department of Biology Zoology Section, Faculty of Science, Ege University, İzmir, Türkiye; 4 Ödemiş Vocational School, Ege University, İzmir, Türkiye; 5 Vaccine Development Application and Research Center, Ege University, İzmir, Türkiye; 6 Department of Vaccine Studies, Institute of Health Sciences, Ege University, İzmir, Türkiye; 7 Department of Parasitology, Faculty of Medicine, Ege University, İzmir, Türkiye; 8 Experimental Eye Research Institute, Ruhr University Bochum, Bochum, Germany; 9 Department of Animal Science, Faculty of Agriculture, Ankara University, Ankara, Türkiye; 10 Department of Animal Science, Faculty of Agriculture, Dicle University, Diyarbakır, Türkiye; 11 Department of Animal Science, Faculty of Veterinary Medicine, Afyon Kocatepe University, Afyonkarahisar, Türkiye; 12 Department of Animal Science, Faculty of Agriculture, Ondokuz Mayıs University, Samsun, Türkiye

**Keywords:** *Anaplasma* spp., *A. ovis*, Sub-cluster, Sequencing, Sheep, Breed

## Abstract

**Purpose:**

Anaplasmosis is a tick-borne disease which is caused by different *Anaplasma* species. Among *Anaplasma* species, *A. ovis* which can infect sheep and goats cause ovine anaplasmosis. In this study, the prevalence of *Anaplasma* spp. was investigated in 31 different breeds of sheep from different regions of Türkiye.

**Method:**

*Anaplasma* spp. was investigated by PCR targeting *MSP-4* gene in blood samples of sheep breeds (n = 366) collected from different regions of Türkiye. Also, some *Anaplasma* spp. positive samples were sequenced for species identification and sub-cluster analyses.

**Results:**

The molecular prevalence of *Anaplasma* spp. was 43.9% (161/366). In Anatolian Merino (n = 10) and Akkaraman (n = 11) breeds, the molecular prevalence of *Anaplasma* spp. reached to 100%. Also, the highest molecular prevalence was detected in Black Sea region by 70% (28/40) and the lowest molecular prevalence was detected in Marmara region by 32% (16/50). While the prevalence of *Anaplasma* spp. was 59.7% in sheep produced in the extensive system, it was found as 39.2% and 9.8% in sheep produced in semi-extensive and intensive systems, respectively. Accordingly, these findings suggest that the production of sheep in the intensive system protects them from tick-borne diseases, which are of great economic importance. According to BLAST results, all sequenced *Anaplasma* spp. positive samples (n = 29) were identified as *A. ovis*. Also, mixed infections were detected in 6 positive samples. The phylogenetic tree constructed by 38 sequence data showed the presence of three different sub-clusters for *A. ovis* (Sub-cluster 1, 2, and 3). Sub-cluster 2 was found as the most prevalent sub-cluster with 42.1% frequency compared to the other sub-clusters.

**Conclusion:**

This study showed that sheep grown in different regions of Türkiye have a high molecular prevalence value for *Anaplasma* spp.

**Supplementary Information:**

The online version contains supplementary material available at 10.1007/s11686-025-01021-2.

## Introduction

Anaplasmosis is a tick-borne disease caused by *Anaplasma marginale*, *A. centrale*, *A. phagocytophilum*, *A. bovis*, *A. ovis*, and *A. platys* [1]. In addition to these *Anaplasma* species, new species, *Candidatus A. odocoilei*, *Ca A. cinensis*, *Ca A. turritanum*, *A. capra, A. boleense, A. phagocytophilum like-1* have also been identified [2, 3]. Among these species, *A. phagocytophilum* and *A. marginale* cause the most significant clinical symptoms in animals. *Anaplasma phagocytophilum* is the causative agent of pasture fever or tick-borne fever (TBF) which is usually associated with high fever, loss of appetite, dullness, and decreased milk production in ruminants including sheep, goat, cattle and deer. *A. marginale*, the most prevalent causative agent of bovine anaplasmosis, causes various clinical signs such as fever, weight loss, calving difficulties, lethargy, icterus, and often death in animals older than 2 years [4]. On the other hand, *A. ovis* causes ovine anaplasmosis, which is usually associated with moderate pathogenicity with mild infections [5, 6]. In addition to sheep, *A. ovis* can infect goats, wild ruminants, rarely cattle and camels [5, 6]. Also, *A. ovis* has been reported in a single human case in Cyprus [7].

Although *Dermacentor andersoni* and *Rhipicephalus bursa* are the main vectors for *A. ovis* [8], *A. ovis* has also been detected in *R. turanicus* [9] and *R. sanguineus* [10] in Türkiye, *Haemaphysalis longicornis* in China [11] and *D. nuttalli* in Mongolia [12].

The prevalence of *A. ovis* in sheep is generally high and a study reported that the prevalence of *A. ovis* reached 91.7% in small ruminants in the northern part of Portugal by PCR targeting the *MSP-4* gene [13]. In a study conducted in Türkiye, *A. ovis* was detected in sheep with a high prevalence of 89.32% using RT-PCR [14]. Genes such as *MSP-4*, *16S*, *gltA*, *groEL* and *OMP*, which are targeted by PCR for diagnosing anaplasmosis are also used in genotyping *A. ovis* strains. For example, a study identified four distinct sub-clusters of *A. ovis* based on *MSP-4* gene sequencing [5].

In Türkiye, several studies have investigated *Anaplasma* spp. in sheep using microscopy, serology, or molecular methods, reporting varying prevalence values. However, fewer studies have been conducted in Türkiye compared to other countries, and they do not provide information on the prevalence of *Anaplasma* spp. in different sheep breeds. Therefore, this study investigated the prevalence of *Anaplasma* spp. in 31 sheep breeds from various regions of Türkiye using PCR targeting the *MSP-4* gene. Additionally, *Anaplasma* spp.-positive samples were sequenced to determine the genotype profile of *Anaplasma* strains through phylogenetic analysis.

## Materials and Methods

### Sheep Breeds

Sheep breeds were identified using the Livestock Information System (HAYBIS) manual from the Republic of Türkiye’s Ministry of Agriculture and Forestry [15]. This system classifies breeds based on tail shape (fine, fat, semi-fat) and breeding origins (imported or domestic) according to regional distribution in Türkiye [16].

A total of 366 sheep blood samples were collected from different regions of Türkiye. The breeds are Akkaraman (n = 11), Morkaraman (n = 24), Kıvırcık (n = 20), Merino (n = 10), Awassi (n = 20), Chios (n = 29), Hamdani (n = 20), Romanov (n = 20), Karayaka (n = 20), Dağliç (n = 15), Kangal Akkaraman (n = 10), Karacabey Merino (n = 10), Anatolian Merino (n = 10), Zom (n = 20), Hemsin (n = 10), Suffolk (n = 10), Eşme (n = 10), Norduz (n = 10), Gökçeada (n = 10), Karya (n = 5), Ramlıç (n = 7), Çine Çaparı (n = 5), Herik (n = 5), Koçeri (n = 4), Polatlı (n = 5), Berrichon du Cher (n = 9), Bafra (n = 5), Ile de France (n = 5), Tahirova (n = 15), Charollais (n = 5), Assaf (n = 5), and East Friesian (n = 2).

## PCR and Sequencing

Genomic DNA was extracted from each blood sample collected from the sheep using a commercial DNA isolation kit (GeneMark, Taichung, Taiwan) according to manufacturer's instructions. A PCR targeting *MSP-4* gene of *Anaplasma* spp. (*A. ovis* and *A. marginale*) was performed on each DNA sample using the primers MSP45 (5′-GGGAGCTCCTATGAATTACAGAGAATTGTTTAC-3′) and MSP43 (5′-CCGGATCCTTAGCTGAACAGGAATCTTTGC-3′) as previously described [17]. Briefly, 25 µl reaction volume consisted of 2 µl template DNA, 1 µl of each primer (10 µM working concentration), 5 µl 5X PCR master mix (GeneMark, Taichung, Taiwan), and 16 µl distilled water. The reaction was performed in a Thermal Cycler (Thermo, PX2) under the following conditions: 30 s initial denaturation at 94 °C, 35 cycles of 94 °C for 30 s, 60 °C for 30 s, 68 °C for 1 min, and a final elongation step at 68 °C for 7 min. The amplified PCR products were visualized on a 1 % agarose gel using Ethidium bromide. To obtain the sequence data for MSP-4 gene, a total of 29 PCR amplicons representing sheep from different regions of Türkiye were processed by the ABI 3500 DNA Sequencer (Applied Biosystems® Sanger Sequencing 3500 Series Genetic Analyzers, Hitachi, Japan) using MSP45 primer, and BLAST analysis was conducted using obtained sequence data for species identification. A phylogenetic tree was constructed by DNAMAN software (Version 5.2.2; Lynnon Biosoft, Que., Canada) using sequence data obtained from this study and reference sequences known to belong to *A. ovis*, *A.marginale*, and *A. phagocytophilum*. During the construction of the phylogenetic tree, genetic distance between *A. ovis* sequences from this study and previously used by Selmi et al. [5] was calculated by the maximum likelihood method and used to construct neighbor-joining trees. Branches of the phylogenetic tree were calculated with bootstrap test with 1000 replicates. Reference sequences including HQ661160.1, KJ700631.1, FJ460454.1, EF067341.1, HQ014384.1, MH292905.1, AY702923.1, KM285217.1, MH292897.1, KC432642.1, KM285218.1, MN094838.1, KY659322.1, FJ460443.1, EU925811.1, KC432643.1, KC432644.1, KM285221.1, KM285220.1, MN094837.1, HQ456350.1, KC432641.1, KM285222.1, AY702924.1, FJ460446.1, JQ621903.1, HQ456348.1, DQ674249.1, DQ674248.1, AY283190.1, KJ512166.1, AY010252.1, AY702920.1, AF428090.1, and AJ580451.1 were used to construct the phylogenetic tree [5].

## Results

**Fig. 1:**
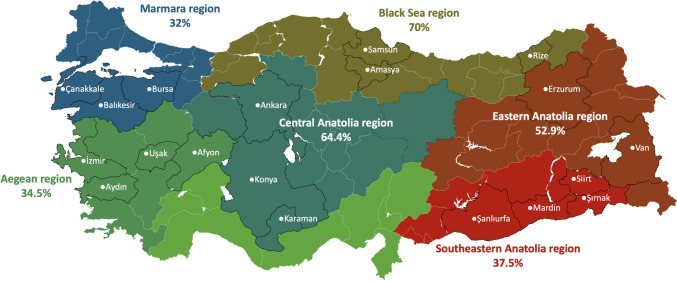
The map shows the provinces where sheep samples were collected as well as the molecular prevalence values of *Anaplasma* spp. in each region of Türkiye

**Fig. 2:**
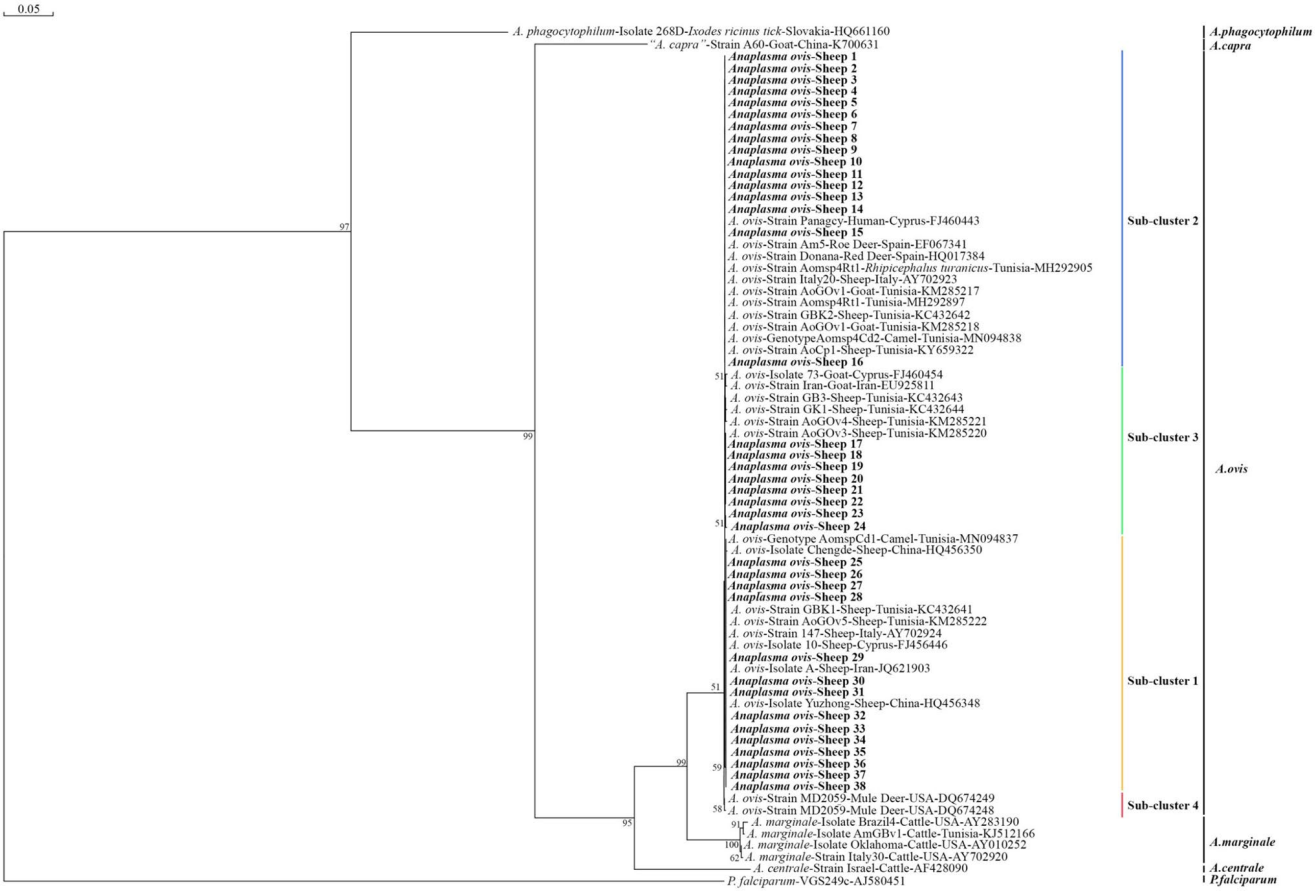
The phylogenetic tree shows that sequenced *Anaplasma* spp.-positive samples are *A. ovis* and *A. ovis* sub-clusters. The examples in bold are from this study and the others are reference examples. A bootstrap value greater than 90% indicates that the branches are highly reliable, while values between 70 and 90% are considered acceptable. Bootstrap values below 70% suggest that the corresponding branches should be interpreted with caution

The molecular prevalence of *Anaplasma* spp. was found to be 43.9% (161/366) among sheep blood samples analyzed. As the molecular prevalence values were examined based on breeds, the molecular prevalence of *Anaplasma* spp. reached 100% in blood samples collected from Anatolian Merino and Akkaraman breeds while *Anaplasma* spp. was not detected in blood samples collected from Karacabey Merino, Koçeri, Berrichon du Cher, Romanov, Tahirova, and East Friesian breeds (Table 1). When molecular prevalence values were analyzed by region, the highest was observed in the Black Sea region at 70% (28/40), while the lowest was in the Marmara region at 32% (16/50) (Figure 1). The prevalence of *Anaplasma* spp. was 59.7% in sheep raised under an extensive system, compared to 39.2% in a semi-extensive system and 9.8% in an intensive system. According to BLAST results, all sequenced *Anaplasma* spp. positive samples (n = 29) were identified as *A. ovis* and showed 100% similarity with reference *A. ovis* isolates. The sequence data of the *A. ovis* positive samples were given in Supplementary File-1. Furthermore, sequence data showed the presence of mixed infections in 6 positive samples (Supplementary File-2). Among the mixed infections, sub-cluster 1, 2 and 3 or sub-cluster 1 and 2 were detected in the same positive samples. Due to mixed infections, although a total of 29 samples were sequenced, the phylogenetic tree was constructed with 38 sequence data. Accordingly, the phylogenetic tree showed the presence of *A. ovis* subcluster 1, 2, and 3 (Figure 2). Sub-cluster 2 was the most prevalent sub-cluster with 42.1% compared to other sub-clusters, followed by sub-cluster 2 with 36.8% and sub-cluster 3 with 21%.

**Table 1:** Molecular prevalence of *Anaplasma spp.* in each breed of sheep and in different regions of Türkiye

Sheep breeds	Number of blood samples tested	Number of *Anaplasma* spp. positive sheep	*Anaplasma* spp. prevalence in each breed (%)	Province	Region	Extensive/intensive livestock production
Kıvırcık	20	10	50	Balıkesir	Marmara region	Extensive
Karacabey Merino	10	–	0	Bursa	Marmara region	Intensive
Merino	10	5	50	Bursa	Marmara region	Intensive
Gökçeada	10	1	10	Çanakkale	Marmara region	Semi-extensive
Awassi	20	18	90	Şanlıurfa	South-eastern Anatolia region	Extensive
Koçeri	4	–	0	Siirt	South-eastern Anatolia region	Extensive
Zom	20	5	25	Mardin	South-eastern Anatolia region	Extensive
Hamdani	20	1	5	Şırnak	South-eastern Anatolia region	Extensive
Anatolian Merino	10	10	100	Ankara	Central Anatolia region	Extensive
Akkaraman	11	11	100	Karaman	Central Anatolia region	Extensive
Kangal Akkaraman	10	6	60	Ankara	Central Anatolia region	Semi-extensive
Polatlı	5	2	40	Ankara	Central Anatolia region	Semi-extensive
Berrichon du Cher	9	–	0	Konya	Central Anatolia region	Intensive
Hemsin	10	8	80	Rize	Black Sea region	Extensive
Karayaka	20	17	85	Samsun	Black Sea region	Extensive
Bafra	5	1	20	Samsun	Black Sea region	Semi-extensive
Herik	5	2	40	Amasya	Black Sea region	Semi-extensive
Norduz	10	4	40	Van	Eastern Anatolia region	Extensive
Morkaraman	24	14	58,3	Erzurum	Eastern Anatolia region	Extensive
Ile de France	5	2	40	İzmir	Aegean region	Semi-extensive
Chios	29	17	58,6	İzmir	Aegean region	Semi-extensive
Dağlıç	15	9	60	Afyon	Aegean region	Extensive
Suffolk	10	1	10	İzmir	Aegean region	Intensive
Eşme	10	1	10	Uşak	Aegean region	Semi-extensive
Karya	5	4	80	Aydın	Aegean region	Extensive
Ramlıç	7	5	71,4	Afyon	Aegean region	Extensive
Çine Çaparı	5	4	80	Aydın	Aegean region	Extensive
Tahirova	15	–	0	İzmir	Aegean region	Intensive
Charollais	5	2	40	İzmir	Aegean region	Intensive
Romanov	20	–	0	İzmir	Aegean region	Intensive
Assaf	5	1	20	İzmir	Aegean region	Semi-extensive
East Friesian	2	–	0	İzmir	Aegean region	Intensive

## Discussion

In Türkiye, *Anaplasma* spp. has been investigated in sheep using microscopy, serological or molecular methods and different prevalence values for *Anaplasma* spp. have been reported. However, the prevalence of *Anaplasma* spp. in different sheep breeds in Türkiye is not well demonstrated. Furthermore, the genotype profile of *Anaplasma* spp. positive samples based on the *MSP-4* gene is also not well known in Türkiye. Accordingly, this study has two different objectives. The first is to find out the molecular prevalence of *Anaplasma* spp. and the second is to reveal the genotype profile of *Anaplasma* spp. samples in Türkiye. In this study, the prevalence value for *Anaplasma* spp. was found as 43.9% in all samples analyzed, while the prevalence value reached 52.9%, 64.4%, and 70% in the Eastern Anatolia, Central Anatolia, and Black Sea regions, respectively. The high prevalence of *Anaplasma* spp. in sheep was consistent with previous studies conducted in Türkiye. For example, in a study on 1,979 samples from small ruminants, the prevalence of *A. ovis* was reported as 41.1% using the reverse line blot (RLB) hybridization assay and 63.3% by PCR. In the same study, the prevalence of *A. ovis* was found to be 68.7% in Central Anatolia by PCR [18]. In another study investigating the presence of *A. ovis* and *A. phagocytophilum* in sheep and goats in Eastern Anatolia, the prevalence of *A. ovis* was found to be 67.35% in sheep by PCR while the prevalence of *A. phagocytophilum* was 18.9% [19]. In a different study conducted in Southeastern Anatolia, the prevalence of *A. ovis* was reported to be 89.32% in sheep using RT-PCR while the prevalence of *A. phagocytophilum* remained at 42.97% [14]. These results, together with our results, show that sheep are frequently bitten by ticks infected with *Anaplasma* spp. in nature. However, this study differs from others. *Anaplasma* spp. was not detected in some sheep breeds (Karacabey Merino, Koçeri, Berrichon du Cher, Tahirova, Romanov and East Friesian) produced in intensive system except Koçeri breed. Accordingly, this study suggests that the production of sheep in the intensive system protects them from tick-borne diseases, which are of great economic importance.

Besides, in this study, all of the sequenced *Anaplasma* spp. positive samples were identified as *A. ovis*, although the PCR used can also detect *A. marginale*. Since *A. marginale* was detected in sheep in previous studies, it implements that increasing the number of samples sequenced in this study would have shown the presence of *A. marginale* among *Anaplasma* spp. positive samples. For example, in previous studies conducted in Pakistan, *A. marginale* which causes bovine anaplasmosis was detected in sheep [20–22] and in one of these studies, *A. ovis* was detected at a higher prevalence rate than *A. marginale*. In parallel with the results of our study, *A. marginale* was not detected among *Anaplasma* spp. positive samples sequenced in this study, but it was reported in cattle in Türkiye [23]. In addition, in a study performed in the Aegean region of Türkiye, *A. marginale* was detected in some tick species, including *Hyalomma marginatum* and *Hyalomma excavatum* [24]. Since *A. marginale* has been detected in sheep and ticks in different countries in Türkiye, it was thought that *A. marginale* should be also investigated in sheep in Türkiye in addition to *A. ovis* during epidemiological studies and clinical cases that may be associated with anaplasmosis in sheep.

According to the genotype profile results of *A. ovis* isolates, the presence of only *MSP-4* based genotype I in sheep was reported in a study conducted in Türkiye [25]. However, 3 different sub-clusters (1, 2 and 3) were identified in this study by a phylogenetic analysis, and it was determined that sub-cluster 2 was more dominant among these sub-clusters. This finding is also important in terms of showing the presence of other sub-clusters of *A. ovis* in addition to genotype I detected in the previous study [25].

## Conclusion

In this study, *Anaplasma* spp. was investigated in different sheep breeds in Türkiye. A very high prevalence value of 43.9% was determined for *Anaplasma* spp. According to the sequence data, all of the sequenced *Anaplasma* spp. positive samples were identified as *A. ovis*. In addition, *MSP-4*-based genotyping demonstrated for the first time the presence of three different subclusters (sub-cluster 1, 2 and 3) in sheep in Türkiye.

## Supplementary Information

Below is the link to the electronic supplementary material.

Supplementary file1 (DOCX 98 KB)

Supplementary file2 (PDF 282 KB)

## Data Availability

No datasets were generated or analysed during the current study.
