# Supplementary material for: Molecular Investigation of Anaplasma spp. and Genotype Profile of A. ovis in Sheep from Different Farms in Türkiye
Source: Acta Parasitol. 2025 Apr 10;70(2):88. doi: 10.1007/s11686-025-01021-2 (PMC11985628; doi:10.1007/s11686-025-01021-2)
Supplement: Supplementary file 1 — Supplementary file1 (DOCX 98 KB) [file 11686_2025_1021_MOESM1_ESM.docx]

***Anaplasma* MSP4 sequence data**

A total of 4 different MSP4 sequence patterns representing 3 sub-clusters were detected among the *Anaplasma* sequence data and are given below.

>Anaplasma_ovis-Sheep_25 (sub-cluster-1)

GTTAGTGGGGCCGTAGTGGCGTCTCCCATGAGTCATGAAGTGGCTTCTGAAGGGAGCGGGGTCATGGGAGGTAGCTTTTATGTGAGTGCGGCTTACAGCCCAGCGTTTCCCTCTGTTACCTCATTCGACATGCGTGAGTCAAGCAGAGAGACCTCGTATGTTAGAGGCTATGACAAGAGTGTTGCAACAATTGATGTGAGTGCGCCAGCAAACTTTTCCAAATCCGGCTACACTTTTGCTTTCTCTAAGAATTTACTCACATCTTTCGACGGCGCTGTGGGATATTCTCTGGGAGGAGCTAGAGTGGAACTAGAAGCAAGCTACAGAAGGTTTGCTACTTTAGCGGACGGGCAGTACGCAAAAAGTGGTGCAGAGTCCCTGGCTGCAATTACTCGCGACGCTGCCATTACTGAGAACAATTACTTTGTGGTCAAAATCGATGAAATCACAAACACTTCAGTCATGCTAAATGGCTGCTATGACGTGTTGCACACAGATTTGCCTGTGTCCCCATATGTGTGTGCCGGAATAGGTGCTAGCTTTGTCGACATTTCTAAGCAAGTAACCACAAAGCTAGCCTACAGGGGCAAGGTTGGAATCAGCTACCAGTTTACTCCAGAAATATCTTTGGTGGTAGGTGGGTTCTACCACGGACTCTTTGACGA

>Anaplasma_ovis-Sheep-1 (sub-cluster 2)

GTTAGTGGGGCCGTAGTGGCGTCTCCCATGAGTCATGAAGTGGCTTCTGAAGGGAGCGGGGTCATGGGAGGTAGCTTTTATGTGAGTGCGGCTTACAGCCCAGCGTTTCCCTCTGTTACCTCATTCGACATGCGTGAGTCAAGCAGAGAGACCTCGTATGTTAGAGGCTATGACAAGAGTGTTGCAACAATTGATGTGAGTGCGCCAGCAAACTTTTCCAAATCCGGCTACACTTTTGCTTTCTCTAAGAATTTACTCACATCTTTCGACGGCGCTGTGGGATATTCTCTGGGAGGAGCTAGAGTGGAACTAGAAGCAAGCTACAGAAGGTTTGCTACTTTAGCGGACGGGCAGTACGCAAAAAGTGGTGCAGAGTCCCTGGCTGCAATTACTCGCGACGCTGTCATTACTGAGAACAATTACTTTGTGGTCAAAATCGATGAAATCACAAACACTTCAGTCATGCTAAATGGCTGCTATGACGTGTTGCACACAGATTTGCCTGTGTCCCCATATGTGTGTGCCGGAATAGGTGCTAGCTTTGTCGACATTTCTAAGCAAGTAACCACAAAGCTAGCCTACAGGGGCAAGGTTGGAATCAGCTACCAGTTTACTCCAGAAATATCTTTGGTGGTAGGTGGGTTCTACCACGGACTCTTTGACGA

>Anaplasma_ovis-Sheep-17(sub-cluster 3)

GTTAGTGGGGCCGTAGTGGCGTCTCCCATGAGTCATGAAGTGGCTTCTGAAGGGAGCGGGGTCATGGGAGGTAGCTTTTATGTGAGTGCGGCTTACAGCCCAGCGTTTCCCTCTGTTACCTCATTCGACATGCGTGAGTCAAGCAGAGAGACCTCGTATGTTAGAGGCTATGACAAGAGTGTTGCAACAATTGATGTGAGTGCGCCAGCAAACTTTTCCAAATCCGGCTACACTTTTGCTTTCTCTAAGAATTTACTCACATCTTTCGACGGCGCTGTGGGATATTCTCTGGGAGGAGCTAGAGTGGAACTAGAAGCAAGCTACAGAAGGTTTGCTACTTTAGCGGACGGGCAGTACGCAAAAAGTGGTGCAGAGTCCCTGGCTGCAATTACTCGCGACGCTGTCATTAATGAGAACAATTACTTTGTGGTCAAAATCGATGAAATCACAAACACTTCAGTCATGCTAAATGGCTGCTATGACGTGTTGCACACAGATTTGCCTGTGTCCCCATATGTGTGTGCCGGAATAGGTGCTAGCTTTGTCGACATTTCTAAGCAAGTAACCACAAAGCTAGCCTACAGGGGCAAGGTTGGAATCAGCTACCAGTTTACTCCAGAAATATCTTTGGTGGTAGGTGGGTTCTACCACGGACTCTTTGACGA

>Anaplasma_ovis-Sheep_23 (sub-cluster-3)

GTTAGTGGGGCCGTAGTGGCGTCTCCCATGAGTCATGAAGTGGCTTCTGAAGGGAGCGGGGTCATGGGAGGTAGCTTTTATGTGAGCGCGGCTTACAGCCCAGCGTTTCCCTCTGTTACCTCATTCGACATGCGTGAGTCAAGCAGAGAGACCTCGTATGTTAGAGGCTATGACAAGAGTGTTGCAACAATTGATGTGAGTGCGCCAGCAAACTTTTCCAAATCCGGCTACACTTTTGCTTTCTCTAAGAATTTACTCACATCTTTCGACGGCGCTGTGGGATATTCTCTGGGAGGAGCTAGAGTGGAACTAGAAGCAAGCTACAGAAGGTTTGCTACTTTAGCGGACGGGCAGTACGCAAAAAGTGGTGCAGAGTCCCTGGCTGCAATTACTCGCGACGCTGTCATTACTGAGAACAATTACTTTGTGGTCAAAATCGATGAAATCACAAACACTTCAGTCATGCTAAATGGCTGCTATGACGTGTTGCACACAGATTTGCCTGTGTCCCCATATGTGTGTGCCGGAATAGGTGCTAGCTTTGTCGACATTTCTAAGCAAGTAACCACAAAGCTAGCCTACAGGGGCAAGGTTGGAATCAGCTACCAGTTTACTCCAGAAATATCTTTGGTGGTAGGTGGGTTCTACCACGGACTCTTTGACGA
