# Supplementary material for: Molecular Investigation of Anaplasma spp. and Genotype Profile of A. ovis in Sheep from Different Farms in Türkiye
Source: Acta Parasitol. 2025 Apr 10;70(2):88. doi: 10.1007/s11686-025-01021-2 (PMC11985628; doi:10.1007/s11686-025-01021-2)
Supplement: Supplementary file 2 — Supplementary file2 (PDF 282 KB) [file 11686_2025_1021_MOESM2_ESM.pdf]

A.ovis Sheep 3, 4, 8, 13, 25, 28, 32 and 34

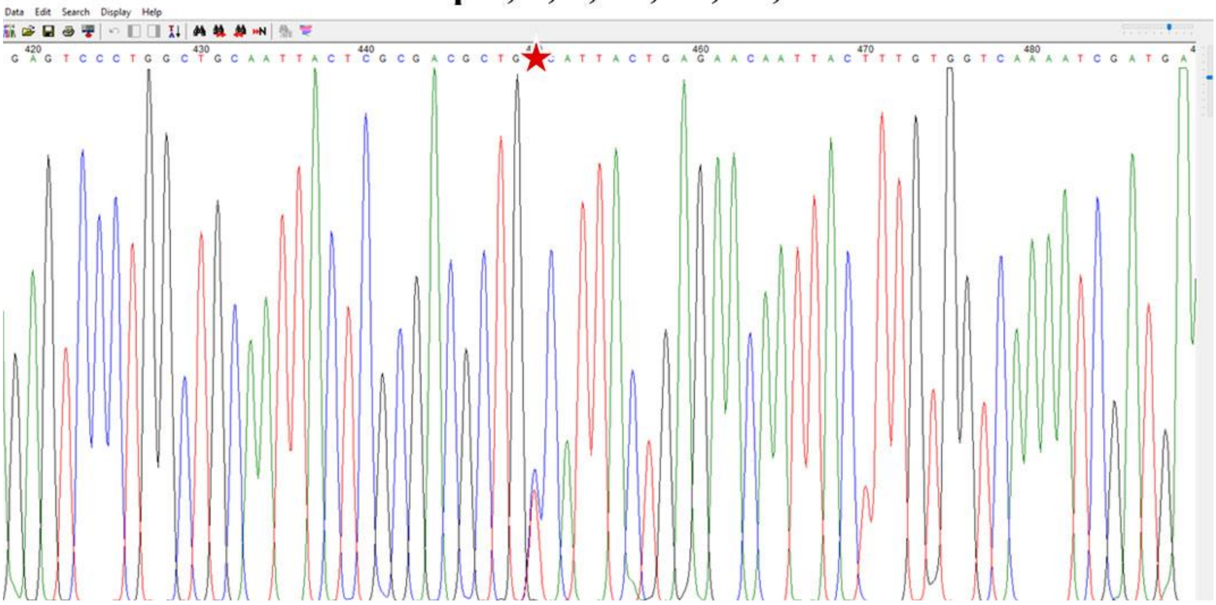

Figure 1. The position where mixed infection was detected was shown with an asterisk (*). The numbers used in the figure heading are related to the *Anaplasma* numbers used in the phylogenetic tree.

A.ovis Sheep 9, 18, 23, 26

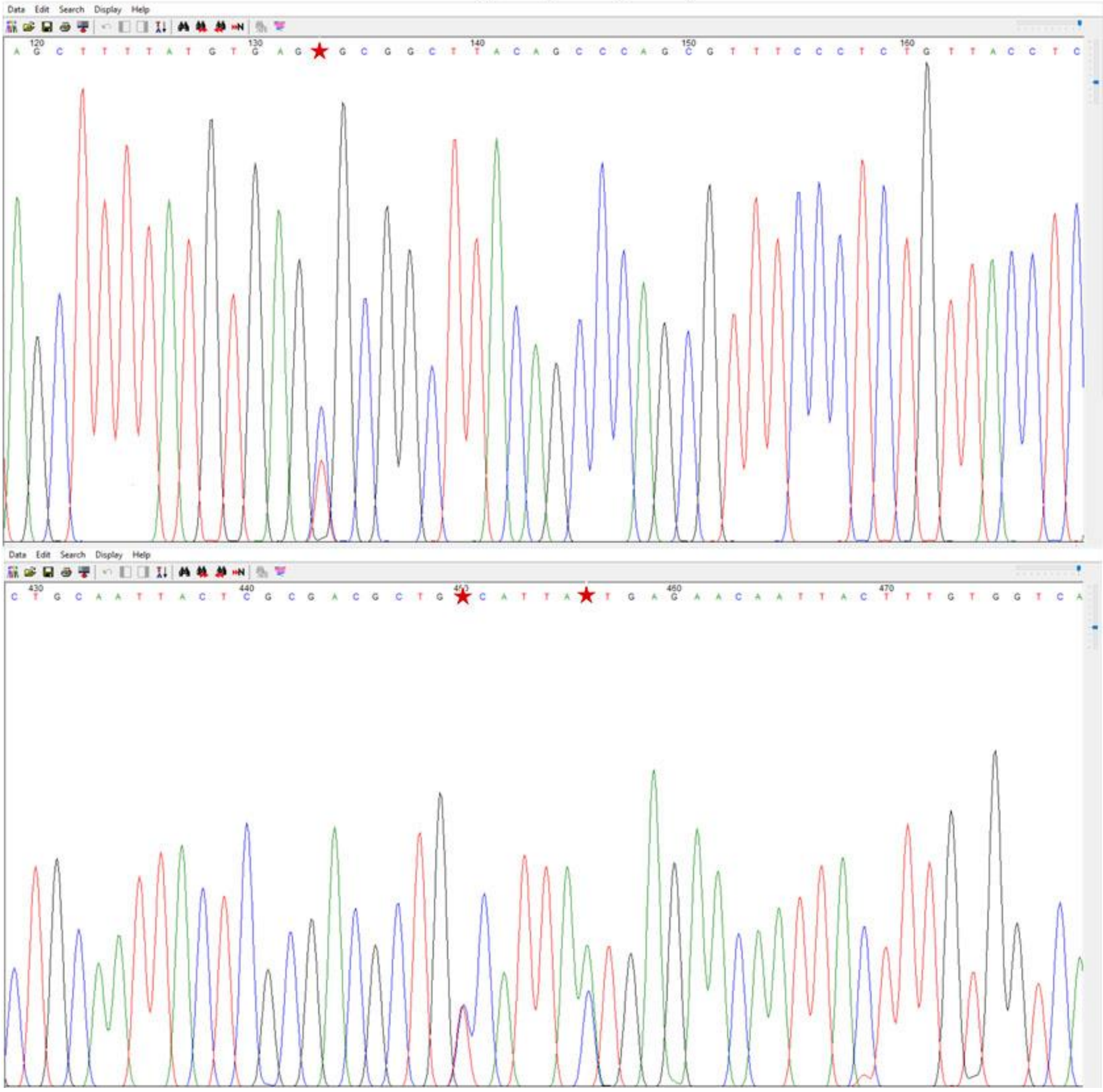

The position where mixed infection was detected was shown with an asterisk (*).The numbers used in the figure heading are related to the *Anaplasma* numbers used in the phylogenetic tree.

A.ovis Sheep 5, 22, 35

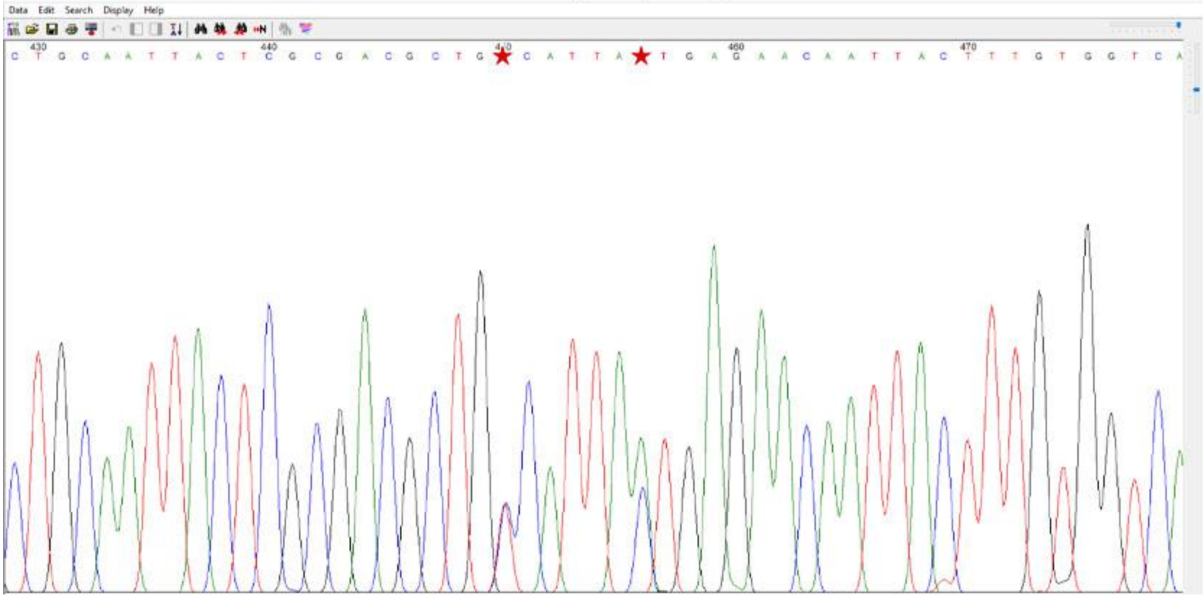

The position where mixed infection was detected was shown with an asterisk (*).The numbers used in the figure heading are related to the *Anaplasma* numbers used in the phylogenetic tree.
